# Multi-Sensor Fusion with Interaction Multiple Model and Chi-Square Test Tolerant Filter

**DOI:** 10.3390/s16111835

**Published:** 2016-11-02

**Authors:** Chun Yang, Arash Mohammadi, Qing-Wei Chen

**Affiliations:** 1 College of Automation, Nanjing University of Science and Technology, Nanjing 210094, China; 311102235@njust.edu.cn (C.Y.); cqw1002@sina.com (Q.-W.C.); 2 Concordia Institute for Information System Engineering, Concordia University, Montreal, QC H3G-1M8, Canada

**Keywords:** fault detection, fault isolation, multi-sensor systems, information fusion, integrated navigation system, interactive multiple models

## Abstract

Motivated by the key importance of multi-sensor information fusion algorithms in the state-of-the-art integrated navigation systems due to recent advancements in sensor technologies, telecommunication, and navigation systems, the paper proposes an improved and innovative fault-tolerant fusion framework. An integrated navigation system is considered consisting of four sensory sub-systems, i.e., Strap-down Inertial Navigation System (SINS), Global Navigation System (GPS), the Bei-Dou2 (BD2) and Celestial Navigation System (CNS) navigation sensors. In such multi-sensor applications, on the one hand, the design of an efficient fusion methodology is extremely constrained specially when no information regarding the system’s error characteristics is available. On the other hand, the development of an accurate fault detection and integrity monitoring solution is both challenging and critical. The paper addresses the sensitivity issues of conventional fault detection solutions and the unavailability of a precisely known system model by jointly designing fault detection and information fusion algorithms. In particular, by using ideas from Interacting Multiple Model (IMM) filters, the uncertainty of the system will be adjusted adaptively by model probabilities and using the proposed fuzzy-based fusion framework. The paper also addresses the problem of using corrupted measurements for fault detection purposes by designing a two state propagator chi-square test jointly with the fusion algorithm. Two IMM predictors, running in parallel, are used and alternatively reactivated based on the received information form the fusion filter to increase the reliability and accuracy of the proposed detection solution. With the combination of the IMM and the proposed fusion method, we increase the failure sensitivity of the detection system and, thereby, significantly increase the overall reliability and accuracy of the integrated navigation system. Simulation results indicate that the proposed fault tolerant fusion framework provides superior performance over its traditional counterparts.

## 1. Introduction

Recent developments in sensor technologies, telecommunication, and navigation systems made *multi-sensor information fusion* an indispensable component of the state-of-the-art integrated navigation systems. Presently, an increasing number of heterogeneous sensors [1] are being used in navigation systems. As a result of recent advancements and the evolution of inertial navigation sensors, global satellite systems and celestial sensor technologies, multi-sensor integrated navigation continues to progress as it is capable of satisfying navigation requirements of high precision, high reliability, and strong autonomy, especially in the fields of aeronautics and astronautics. The paper develops a multi-sensor fusion framework to meet the requirements of long-time and high-precision navigation of carriers and moving objects such as long-range missiles, naval vessels, long-range bombers, and High Altitude Long-Endurance Unmanned Aerial Vehicles (HALE-UAVs). Especially in the latter case (i.e., HALE-UAV), it is necessary to continuously provide all-time and all-weather high-precision motion parameters over a long period of time. Consequently, the incorporation of an efficient and advanced multi-sensor fusion framework is both critical and essential to achieve the high-performance, reliability, and real-time requirements of modern navigation systems. Besides, multi-sensor failures can occur at the same time for example due to external environment disturbances; therefore, it is practically hard to perform failure detection and isolation with the required high precision using traditional methodologies. The multi-sensor and fault-tolerant fusion framework proposed in this paper is designed to address this issue. The proposed framework can detect multi-sensor failures and isolate the failed sub-system adaptively, and as long as the faults of the subsystem disappear, the system will return to the optimal integrated mode.

Multi-sensor information fusion in integrated navigation, typically comprises a reference navigation system corrected using measurements from other constituent sub-systems. The reference system commonly consists of an Inertial Navigation System (INS) or Strap-down Inertial Navigation System (SINS), which are sophisticated electromechanical systems that continuously provide, via the dead reckoning, position, orientation and velocity of a moving object (e.g., aircraft, missile or ship), using motion (accelerometers) and rotation (gyroscopes) sensors. Depending on different specifications of the application at hand, such as the environment, dynamics, budget and accuracy requirements, INS is combined with other navigation sensors in the fusion framework, including, among others: (i) the Global Positioning System (GPS), which is a commonly used navigation system for providing three-dimensional position and velocity information with high acceptable accuracy; (ii) the Bei-Dou2 (BD2) navigation system [2,3] which is a Chinese global satellite navigation system consisting of 35 satellites; and (iii) the Celestial Navigation System (CNS) [4], which is an autonomous navigation system based on celestial observation by Sun/star sensors, which is capable of providing attitude and position information without accumulating error over time. Recently, there has been a surge of interest [4,5,6,7,8] in the development and implementation of INS/GPS/CNS integrated navigation systems. An INS operates continuously in an autonomous fashion with a high data rate and high precision during a short time span (i.e., relatively small short-term errors); therefore, it is at the core of an integrated navigation system. However, the accuracy of INS degrades over time as the position error increases unboundedly (error is accumulated through the INS algorithm over time) [9]. In comparison to the INS, the GPS (and similarly BD2) has some drawbacks such as possessing low update frequency together with high amplitude observation noise. Besides, its navigation accuracy and integrity significantly degrades in adverse circumstances [10,11,12]. Finally, the CNS has its own drawbacks including low update rate besides being vulnerable to weather circumstances [4]. Consequently, each of these navigation sensors has disadvantages in stand-alone mode, rendering them incapable of meeting the long-time and high-performance requirements of state-of-the-art navigation systems when used in stand-alone mode. Integration of the INS, CNS, GPS and BD2 allows us to make full use of the complementary advantages among each navigation sub-system to greatly improve the precision and reliability of the overall navigation system. Especially when the system is used in long-range missiles, naval vessels, long-range bombers and HALE-UAV applications, the need for high reliability and fault-tolerant performance justifies the costs associated with the extra complexity of the procedure.

Multi-sensor information fusion is the key component for optimal and efficient integration of these navigation sensors and is the focus of this paper. The above mentioned navigation sensors are highly complementary, and their integration through an appropriate multi-sensor fusion framework results in the utilization of their complementary advantages and the improvement of the navigation system’s overall reliability and accuracy. Despite recent advancements in this regard, on the one hand, fault detection and integrity monitoring are critically required for multi-sensor integration architectures, an issue that has been overlooked in the recent literature. On the other hand, the design of an integrated navigation system is extremely constrained especially when no information regarding the system’s error characteristics (e.g., error covariances) is available. The paper focuses on these problems and proposes an innovative fault tolerant multi-sensor information fusion framework applied in the INS/GPS/BD2/CNS integrated navigation system for improving the accuracy and reliability of such systems. In order to achieve this goal, fault detection and isolation play a crucial role [13]. First, we briefly review relevant research works in this context to better motivate the contributions of the paper to be outlined after this short literature review.

It is well-known that the chi-square test [14] is the conventional and commonly used failure detection methodology to monitor dynamical systems and estimation algorithms developed based on the Kalman Filter (KF). The chi-square test belongs to the class of statistical hypothesis-testing mechanisms and is commonly used to examine whether the assumed mean and covariance matrices match the actual settings or not. For instance, [14] proposed to utilize a setup based on two state propagators, alternatively selected based on the Kalman filter’s output, to prevent the risk of using a corrupted propagator for failure detection purposes resulting in an increased failure detection sensitivity. Traditionally, however, the chi-square test is applied for single failure detection and isolation by setting a threshold to judge whether the system is failed or not [15,16]. For instance, [17] employed fuzzy logic and weighted average to deal with the threshold and to reduce the percentage of incorrect alarm effectively. However, in the INS/GPS/BD2/CNS integrated navigation system, there might be multi-sensor failures at the same time, and the failure type may also be different; therefore, it is hard to perform failure detection and isolation with traditional methods. In [18], a data fusion method with a supervised estimation process is proposed to address this issue. Caron et al. in [19] proposed to apply the method developed in [18] into integrated a GPS and Inertial Measurement Unit (IMU) multisensory fusion system. Through introducing contextual information in the fusion system, the proposed detection solution of [19] is capable of rejecting false information to increase the reliability of the multi-sensor fusion algorithm. In [20], a fault-tolerant multi-sensor fusion algorithm is developed specifically for the SINS/GPS/BD2/CNS integrated navigation system. In this work, the two state propagators’ chi-square test is used as the failure detection method which is then combined with the fusion strategy of [18] to effectively detect and isolate multi-sensor failures. However, in scenarios where the accuracy of the inertial measurement units in the SINS is low, the aircraft manoeuvre will damage the accuracy of the SINS, thereby, reducing the failure detection sensitivity. The interacting multiple model (IMM) estimation algorithms [21,22,23] are, therefore, designed to overcome this difficulty by incorporating a set of candidate models to represent different uncertainties under realistic conditions. Research on the fault detection and isolation problem with the IMM algorithm has attracted a recent surge of interest [24,25,26,27,28,29]. To the best of our knowledge, however, few of these aforementioned methodologies can detect multi-failures at the same time effectively. The paper addresses this gap and develops an improved multi-sensor fault tolerant fusion framework applied in the INS/GPS/BD2/CNS integrated navigation system.

In this paper and to improve the reliability and fault-tolerant capability of the aircraft navigation system, we propose an innovative fault-tolerant integrated navigation framework via fusion of SINS, GPS, BD2, and CNS sensors. The problem considered here is a real-world challenge which has not yet been addressed properly. The goal of the proposed fusion framework is to improve the accuracy and reliability of the overall system and the main contribution is to look at the system as whole and the combination (fusion) strategy proposed to solve this real-world problem we faced. Noting that the aircraft manoeuvre will damage the accuracy of the inertial measurement unit and a precisely known model of the system is hard to be achieved, we define a two level process noise covariance matrix as the system uncertainty under low and high manoeuvres. With the combination of the IMM filter, the probabilities of each model will be adjusted adaptively through a modified measurement update step. Thus, the accuracy of the state propagator will be increased. On the other hand, in order to improve the failure sensitivity of the detection solution, we combine the state chi-square test with two state propagators. These two state propagators are alternatively reactivated based on the information they receive from the fusion filter to increase the failure sensitivity of the detection system. By choosing a suitable time interval between re-setting the state propagators and using them for failure detection, the risk of having corrupted information will be avoided (minimized). By incorporating the value of the state chi-square test obtained from each sub-system, we propose an improved fusion method to isolate potential failures. Furthermore, we calculate the appropriate likelihood of the measurement to update the probability of each model. With the combination of the IMM Kalman Filter (IMM-KF) and the multiple sensor fusion method, we increase the failure sensitivity of the detection algorithm and, thereby, increase the overall accuracy of the integrated navigation system. The implementation of the proposed multi-sensor information fusion framework has the potential to significantly enhance the robustness, real-time reliability, precision, and performance of the underlying navigation system in comparison to its stand-alone navigation counterparts. In summary, the key contributions of the paper are as follows:

- First, we define a two level process noise covariance as the system model uncertainties under low and high manoeuvres; with the idea of the IMM algorithm, we use the IMM to predict estimates without the measurement update as the fault detection reference. Thus, the uncertainty of the system will be adjusted by the model probabilities adaptively with the aircraft manoeuvre.
- To address the problem of incorporating corrupted state propagators for fault detection purposes, the paper designs two state propagators’ chi-square fault detection procedure for the SINS/GPS/BD2/CNS integrated navigation system. We develop two IMM predict estimates, running in parallel without an update step, as the state propagators. State propagators are alternatively reactivated based on the information they receive from the fusion filter to increase the fault sensitivity of the detection solution.
- A fusion strategy is then investigated to realize the fault isolation. In order to use the appropriate measurement to update the model probabilities of the IMM filter, we take advantage of the fault detection information of each measurement and utilize the measurement with the minimum fault detection value to update the model probabilities of the IMM filter. As a result, we can update model probabilities with the optimal measurement, therefore, further increasing the accuracy of the multi-sensor fusion framework.

**Figure 1:**
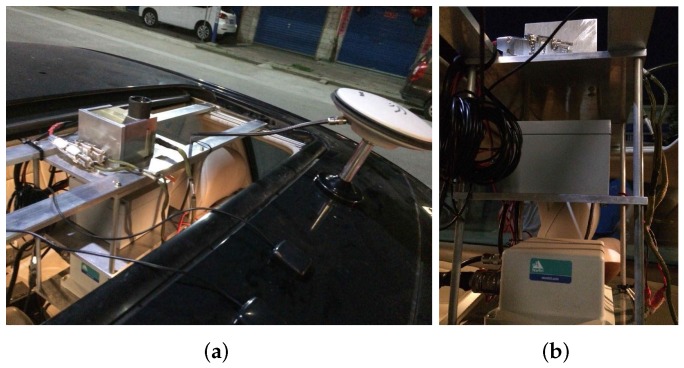
Hardware design of the INS/GPS/Bei-Dou2(BD2)/Celestial Navigation System (CNS) integrated navigation system. (**a**) Hardware view1; (**b**) Hardware view2.

Finally, it is worth mentioning that the proposed fault-tolerant, multi-sensor fusion framework is developed to be implemented, and applied in the real-world settings. In particular as illustrated in Figure 1, we have completed the hardware design of the INS/GPS/BD2/CNS integrated navigation system and now are working to debug the system and implement the proposed fusion framework.

The rest of the paper is organized as follows. Section 2 formulates the problem and presents the proposed multi-sensor system architecture together with the state and measurement models. Section 3 describes the proposed state estimation methodology and presents the proposed failure-tolerant integrated navigation framework. Numerical simulations are performed to verify the performance of the proposed integrated navigation algorithm and presented in Section 4. Finally, Section 5 concludes the paper.

## 2. Multi-Sensor System Architecture

In this section, first we define the state-model of the main system and then form the measurement models for the other involved sub-systems. The SINS is used as the primary/major navigation sub-system while the GPS, the BD2, and the CNS form the remaining three sub-systems. With the Dead-Reckoning (DR) algorithm [30], we can obtain the position, velocity and the attitude information output from the SINS. The error model of the SINS is a psi-angle error model [30,31] given by:

(1) $$\begin{array}{ccc} \delta \dot{r} & = & -w_{en}\times \delta r+\delta v \end{array}$$

(2) $$\begin{array}{ccc} \delta \dot{v} & = & C\delta f^{b}+\left[\begin{array}{ccc} -\frac{g}{r_{e}} & 0 & 0\\ 0 & -\frac{g}{r_{e}} & 0\\ 0 & 0 & \frac{2g}{r_{e}+h} \end{array}\right]\delta r-\left[\Psi \times \right]f-\left[(w_{\text{en}}+2w_{\text{ie}})\times \right]\delta v \end{array}$$

(3) $$\begin{array}{ccc} \dot{\Psi } & = & -\left[(w_{\text{en}}+2w_{\text{ie}})\times \right]\Psi +C\delta w_{\text{ib}}^{b}, \end{array}$$

where δr is the position error vector; δv is the velocity error vector; **Ψ** is the attitude error vector; $w_{en}$ is the true rate of the local geographic frame with respect to the Earth frame; $w_{ie}$ is the Earth rate vector with respect to the inertial frame; f is the specific force vector; $\delta f^{b}$ is the accelerometer error vector in the body frame; $\delta w_{\text{ib}}^{b}$ is the gyro error vector in the body frame; C is the direction cosine matrix; *g* is the gravity; $r_{e}$ is the radius of the Earth; and term [A×] is the skew-symmetric matrix form (also known as the cross-product form [30]) of vector A. The biases of the accelerometer and the gyroscope are modelled by a first-order Gauss–Markov process with time constants of *τ*. Consequently, the state vector includes fifteen states, i.e., three inertial error states in position, velocity, attitude, accelerometer bias and gyro bias and is denoted by:

(4) $$\begin{array}{c} \dot{x}={\left[\delta r_{x},\delta r_{y},\delta r_{z},\delta v_{x},\delta v_{y},\delta v_{z},\Psi_{x},\Psi_{y},\Psi_{z},\varepsilon_{ax},\varepsilon_{ay},\varepsilon_{az},\varepsilon_{gx},\varepsilon_{gy},\varepsilon_{gz}\right]}^{T}, \end{array}$$

where superscript T denotes the transpose operator. Note that term $\varepsilon_{a}$ is the bias vector corresponding to the accelerometer, and term $\varepsilon_{g}$ is the gyroscope’s bias vector. This completes our discussion on the state model; next, we present the measurement models of different sub-systems.

### System Measurement Models

In order to correct the errors of the SINS sub-system, external measurements are required to calibrate the SINS. As stated previously, in this work we use GPS, BD2 and CNS as the measurement sub-systems, which are then combined with the SINS output to produce the SINS/GPS, SINS/BD2 and SINS/CNS integrated navigation sub-systems. Below, we present details corresponding to each of these three sub-systems:

(1) SINS/GPS integrated navigation sub-system: We use the position information outputted from the GPS to calibrate the SINS, therefore, the measurement equation is defined as $z_{\text{GPS}}=H_{\text{GPS}}x+\xi_{\text{GPS}}$, where x is the state vector, $\xi_{\text{GPS}}$ is the measurement noise vector and $z_{\text{GPS}}$ is the error vector between the SINS and the GPS given by:
  (5) $$\begin{array}{ccc} z_{\text{GPS}} & = & \left[\begin{array}{c} r_{{\text{SINS}}_{x}}-r_{{\text{GPS}}_{x}}\\ r_{{\text{SINS}}_{y}}-r_{{\text{GPS}}_{y}}\\ r_{{\text{SINS}}_{z}}-r_{{\text{GPS}}_{z}} \end{array}\right]. \end{array}$$
  Finally, the SINS/GPS observation model is given by $H_{\text{GPS}}=\left[I_{3\times 3}\;\;\;0_{3\times 12}\right]$, where $I_{3\times 3}$ is a (3×3) identity matrix, and $0_{3\times 12}$ is a (3×12) matrix of all zero elements.
(2) SINS/BD2 integrated navigation sub-system: In this configuration, we use the position information provided by the BD2 sub-system to calibrate the SINS which results in the following measurement equation $z_{\text{BD}2}=H_{\text{BD}2}x+\xi_{\text{BD}2}$, where $\xi_{\text{BD}2}$ is the measurement noise vector, and $z_{\text{BD}2}$ is the error vector between the SINS and the BD2 which is given by:
  (6) $$\begin{array}{ccc} z_{\text{BD}2} & = & \left[\begin{array}{c} r_{{\text{SINS}}_{x}}-r_{{\text{BD}2}_{x}}\\ r_{{\text{SINS}}_{y}}-r_{{\text{BD}2}_{y}}\\ r_{{\text{SINS}}_{z}}-r_{{\text{BD}2}_{z}} \end{array}\right]. \end{array}$$
  Similar to the SINS/GPS scenario, the SINS/BD2 observation model is $H_{\text{BD}2}=\left[I_{3\times 3}\;\;\;0_{3\times 12}\right]$.
(3) SINS/CNS integrated navigation sub-system: In this scenario, the attitude information calculated by the CNS is used to calibrate the SINS. The measurement equation is, therefore, given by $z_{\text{CNS}}=H_{\text{CNS}}x+\xi_{\text{CNS}}$, where $z_{\text{CNS}}$ is the error vector between the SINS and the CNS, which is together with the observation model of this sub-system given by:
  (7) $$\begin{array}{ccc} z_{\text{CNS}} & = & \left[\begin{array}{c} \gamma_{\text{SINS}}-\gamma_{\text{CNS}}\\ \theta_{\text{SINS}}-\theta_{\text{CNS}}\\ \varphi_{\text{SINS}}-\varphi_{\text{CNS}} \end{array}\right] \end{array}$$
  (8) $$\begin{array}{ccc} H_{\text{CNS}} & = & [0_{3\times 6}\;\;\;C_{3\times 3}\;\;\;0_{3\times 6}], \end{array}$$
  where $\xi_{\text{CNS}}$ is the measurement noise vector, and $C_{3\times 3}$ is the conversion matrix [4]. This completes our presentation of the measurement models for the three involved sub-systems.

**Figure 2:**
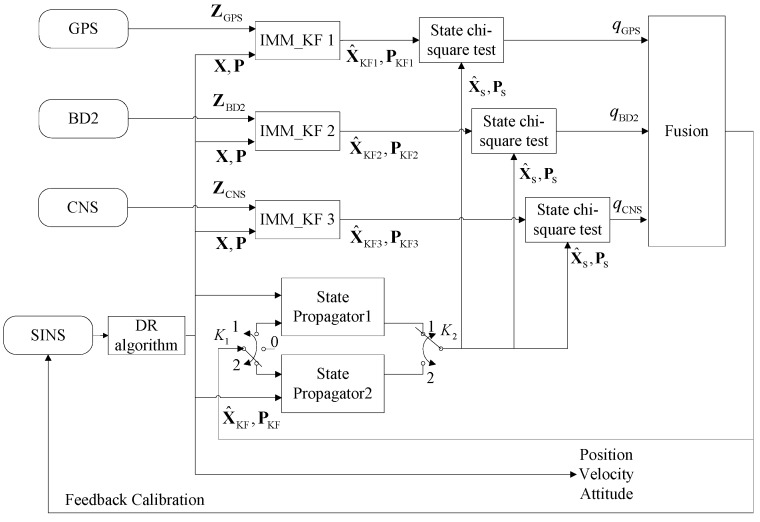
System architecture. SINS, Strap-down Inertial Navigation System; DR, Dead-Reckoning.

Figure 2 illustrates the architecture of the proposed integrated navigation system, including the four aforementioned sub-systems. In this proposed framework, we use the sensory data obtained from the SINS to calculate the parameters of the system model (Equations (1)–(3)) at every SINS sample interval using the DR algorithm. Then, we can predict the system’s state and its corresponding covariance matrix denoted by X and P in Figure 2, respectively, using the IMM-predict process described in detail in Section 3. The IMM predicted estimates without incorporating the measurement update step are used as two state propagators running in parallel. These two state propagators send the state estimate $X_{S}$ and its covariance matrix $P_{S}$ to every state chi-square test block, which is then used as the detection reference. At every measurement update interval, each measurement sub-system sends its measurement value to the IMM_KF block to update the state estimate and its associated covariance matrix. Each sub-system, then, computes the updated state estimate, denoted by $X_{KF,i}$ in Figure 2 (where *i* stands for the corresponding measurement sub-system), and its associated covariance matrix denoted by $P_{KF,i}$ and forwards these statistics to its state chi-square test block for failure detection. Each failure detection block computes the failure detection value $q_{*}$ corresponding to its measurement sub-system and communicates it to the fusion process to perform failure isolation and data fusion tasks. In order to use the appropriate measurement to update the model probabilities of the IMM filter, we take advantage of the fault detection information of each measurement and use the measurement with the minimum fault detection value to update the model probabilities of the IMM filter. As a result, we can update model probabilities with the most favourable measurement, therefore, further increasing the accuracy of the multi-sensor fusion framework. At every feedback calibration interval, we use the fusion data to calibrate the SINS errors. In order to increase the accuracy of the state propagators, we use the fusion state, denoted by ${\hat{X}}_{\text{KF}}$ in Figure 2, and its associated covariance matrix, denoted by ${\hat{P}}_{\text{KF}}$, to reset the state estimates $X_{S}$ and its covariance $P_{S}$. However, because there is usually a time interval between the time when a failure happens and when it is detected, the possibility exists that the state propagator may be contaminated by the fusion data if an undetected failure has already happened. To avoid this problem, we switch $K_{1}$ to Position 2 to reset the State Propagator 2; at the same time, we switch $K_{2}$ to Position 1 to use the State Propagator 1 as the failure detection reference. At the next switching time step, we switch $K_{1}$ to Position 1 to reset the State Propagator 1; at the same time, we switch $K_{2}$ to Position 2 to use the State Propagator 2 as the failure detection reference. With this strategy, a newly reset state propagator does not work as the failure detection reference right away. Thus, we can make sure that no failure occurred before resetting the state propagator; this will be discussed in detail later in Section 3.2. This completes the presentation of the state and observation models. Before further discussions, below, we briefly explain the theoretical challenges encountered in the development of the proposed fusion framework:

(1) Incorporation of an appropriate measurement to update the modal probabilities of the IMM filter is the first theoretical challenge faced in development of the proposed multi-sensor fusion framework. To address this challenge, we combine the failure detection algorithm and the IMM filter and utilize fault detection values to perform the update step of the IMM filter.
(2) The second theoretical challenge is how to isolate a failed measurement adaptively. The value of the state chi-square test obtained from each sub-system is incorporated in the fusion framework to overcome this challenge and isolate a failed measurement.
(3) The third theoretical challenge is the selection of the failure threshold. It is theoretically difficult to find the optimum threshold that minimizes the false alarm rate and at the same time provides optimal accuracy for detecting soft failures. If the threshold is too small, the false alarm rate will be increased, on the other hand with a large threshold, it would be difficult to detect soft failures. To address this issue, we set the threshold as a function of the fault detection values with a bottom boundary and a top boundary. In other words, when the detection value is below the bottom boundary, the sensor’s measurement is accepted, and when the detection value exceeds the top boundary, it is rejected. When the detection value is between the two boundaries, the fault degree of the measurement is computed as a function of the detection value (fuzzy-based solution).
(4) The forth challenge is how to isolate a failure adaptively based on the proposed failure detection framework. Here, because the detection threshold is defined as a function of the detection value, it is not possible to isolate a failed sensor simply by comparing it with a predefined threshold. To address this challenge, first, we define the validation probability of each measurement as a function of its corresponding failure detection values. Then, an adaptive weight for each sub-system is calculated based on its validation probability. Finally, the failure detection is performed adaptively through the calculated weights.

## 3. Failure-Tolerant Integrated Navigation Framework

As stated previously, [14] proposed an efficient way to improve the failure sensitivity of the detection algorithm by the incorporation of two state propagators. However, when this method is being incorporated into an integrated navigation system, the failure sensitivity of the detection solution degrades significantly due to difficulties in maintaining complete/precise knowledge of the state model. An efficient way to take this into account is to consider a nominal model affected by uncertainty; however, the uncertainty will change under different levels of manoeuvres. In order to improve failure detection sensitivity due to model uncertainty, we propose to combine the IMM filter and chi-square test (the failure detection algorithm). Utilization of the IMM algorithm makes it possible to tune an appropriate value for the covariance matrix of the state-noise in order to maintain acceptable estimation accuracy. However, when the IMM filter is applied to the failure detection algorithm, because it updates the probability of each model using received measurements, the detection results could be adversely affected by failed measurements. In other words, it is hard to detect soft failures of the measurements. To solve this problem, we refer to the method proposed in [14], where two state propagators were used as test references. These two state propagators are alternatively reset based on the data provided by the fusion result to increase their accuracy. By choosing a suitable time interval between resetting a state propagator and using it for the failure detection reference, the risk of updating each model by failed measurements will be avoided, and thereby, the failure detection sensitivity will be increased. In the development of the proposed IMM-based fault detection/fusion framework, we use the following difference equations:

(9) $$\begin{array}{ccc} x_{k+1} & = & Fx_{k}+\omega_{k} \end{array}$$

(10) $$\begin{array}{ccc} z_{k} & = & Hz_{k}+\xi_{k}, \end{array}$$

where (k≥1) is the time index, $x_{k}$ and $\omega_{k}$ represent the state vector and process noise vector, respectively, and $z_{k}$ and $\xi_{k}$ are the measurement vector and the measurement noise vector, respectively. Vectors $\omega_{k}$ and $\xi_{k}$ are zero-mean Gaussian white sequences and have zero cross-correlation, i.e., $E\left\{w_{k}w_{i}^{T}\right\}=Q_{k}\delta_{ik}$, $E\left\{\xi_{k}\xi_{i}^{T}\right\}=R_{k}\delta_{ik}$, and $E\{w_{k}\xi_{i}^{T}\}=0$, for all *i* and *k*, where $Q_{k}$ is the process noise covariance matrix at time *k*, and $R_{k}$ is the measurement noise covariance matrix at time *k*. The symbol $\delta_{ij}$ stands for the Kronecker delta function. Details of combining the IMM-KF and state chi-square test will be presented in the following sub-sections, respectively.

### 3.1. Local IMM-KF Estimation Algorithm

**Figure 3:**
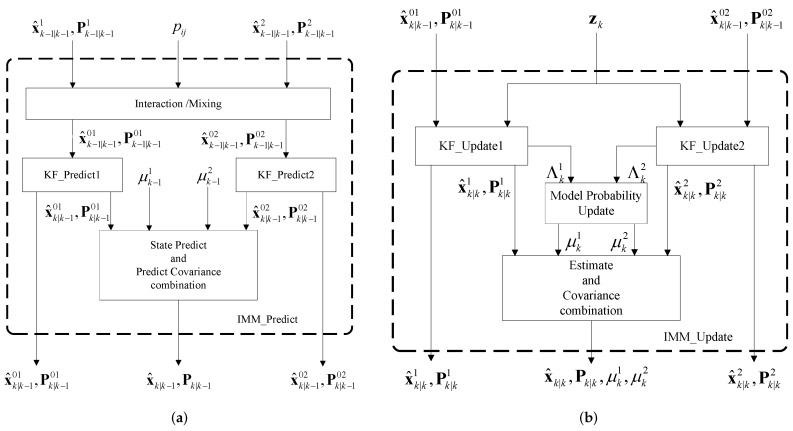
(**a**) Flowchart for Interacting Multiple Model (IMM)-predict; (**b**) Flowchart for IMM-update.

The proposed SINS/GPS/BD2/CNS integrated navigation system consists of three local IMM-KFs as shown in Figure 2. Each filter performs prediction and update steps sequentially based on GPS, BD2 or CNS measurements. One iteration of the IMM-KF consists of two steps, i.e., IMM-predict and IMM-update. Figure 3a illustrates different steps of the IMM-predict as described below:

- Interaction/mixing step: The mixing probability $\mu_{k}^{i|j}$ for models $M^{i}$ and $M^{j}$ are calculated as ${\bar{c}}^{j}=\sum_{i=1}^{n}p_{ij}\mu_{k-1}^{i}$ and $\mu_{k}^{i|j}=\frac{1}{{\bar{c}}^{j}}p_{ij}\mu_{k-1}^{i}$, where $\mu_{k-1}^{i}$ is the probability of model $M^{i}$ being in effect at the time step (k−1), ${\bar{c}}^{j}$ is a normalization factor, and $p_{ij}$ is the model transition probability matrix which is given by $p_{ij}=\left[\begin{array}{cc} \pi_{11} & \pi_{12}\\ \pi_{21} & \pi_{22} \end{array}\right]$. The mixed inputs (i.e., state estimates and their corresponding covariance matrices) for each filter are computed as follows
  (11) $$\begin{array}{cc} {\hat{x}}_{k-1|k-1}^{0j} & =\sum_{i=1}^{2}\mu_{k}^{i|j}{\hat{x}}_{k-1|k-1}^{i}\\ P_{k-1|k-1}^{0j} & =\sum_{i=1}^{2}\mu_{k}^{i|j}\left\{P_{k-1|k-1}^{i}+\left[{\hat{x}}_{k-1|k-1}^{i}-{\hat{x}}_{k-1|k-1}^{0j}\right]{\left[{\hat{x}}_{k-1|k-1}^{i}-{\hat{x}}_{k-1|k-1}^{0j}\right]}^{T}\right\},\; \end{array}$$
  where j=1,2 refers to the model number.
- Mode-matched prediction step: In this step, the KF matched to mode *j*, for (1≤j≤2), computes the predicted state estimate and its corresponding covariance matrix as ${\hat{x}}_{k|k-1}^{0j}=F^{j}{\hat{x}}_{k-1|k-1}^{0j}$ and $P_{k|k-1}^{0j}=F^{j}P_{k-1|k-1}^{0j}{\left[F^{j}\right]}^{T}+Q^{j}$.
- Global prediction step: The predicted statistics of the mode-matched KFs are combined as follows to form the global predicted state estimate and its corresponding covariance matrix
  $$\begin{array}{c} x_{k|k-1}=\sum_{j=1}^{2}\mu_{k-1}^{j}{\hat{x}}_{k|k-1}^{0j}\;\text{and}\;\;P_{k|k-1}=\sum_{j=1}^{2}\mu_{k-1}^{j}\left\{P_{k|k-1}^{0j}+\left[x_{k|k-1}^{0j}-{\hat{x}}_{k|k-1}\right]{\left[x_{k|k-1}^{0j}-{\hat{x}}_{k|k-1}\right]}^{T}\right\}. \end{array}$$

This completes the prediction step (referred to as IMM-predict) of the IMM-KF filter. Figure 3b illustrates different steps of the IMM-update algorithm as described below:

- Mod-matched KF update (KF-update): The KF matched to mode *j*, for (1≤j≤2), updates the following parameters $K^{j}=P_{k|k-1}^{0j}{\left[H^{j}\right]}^{T}{\left[R^{j}\right]}^{-1}$; $\zeta_{k}^{j}=z_{k}-H^{j}{\hat{x}}_{k|k-1}^{0j}$; $S_{k}^{j}=H^{j}P_{k|k-1}^{0j}{\left[H^{j}\right]}^{T}+R^{j}$; ${\left[P_{k|k}^{j}\right]}^{-1}={\left[P_{k|k-1}^{0j}\right]}^{-1}+{\left[H^{j}\right]}^{T}{\left[R^{j}\right]}^{-1}H^{j}$, and; $\Lambda_{k}^{j}=N\left(\zeta_{k}^{j};0,S_{k}^{j}\right)$, where N(a;b,c) denotes the distribution of a Gaussian random variable *a* with mean *b* and variance/covariance *c*. Term $\Lambda_{k}^{j}$ is the likelihood function corresponding to measurement *j*. Note that, each of the three IMM-KFs use their specific observation ($z_{\text{GPS}},z_{\text{BD}2}$, or $z_{\text{CNS}}$) instead of $z_{k}$.
- Model probability update: The probabilities of model $M^{j}$ at time step *k* are calculated as $c=\sum_{j=1}^{2}\Lambda_{k}^{j}{\bar{c}}^{j}$ and $\mu_{k}^{j}=\frac{1}{c}\Lambda_{k}^{j}{\bar{c}}^{j}$, where *c* is a normalization factor.
- Estimate and covariance combination: In the final stage of the IMM-update algorithm, the combined estimate for the state mean and covariance matrix is computed as:
  $$\begin{array}{c} {\hat{x}}_{k|k}=\sum_{j=1}^{2}\mu_{k}^{j}{\hat{x}}_{k|k}^{j}\;\text{and}\;\;P_{k|k}=\sum_{j=1}^{2}\mu_{k}^{j}\left\{P_{k|k}^{j}+\left[{\hat{x}}_{k|k}^{j}-{\hat{x}}_{k|k}\right]{\left[{\hat{x}}_{k|k}^{j}-{\hat{x}}_{k|k}\right]}^{T}\right\}. \end{array}$$

This completes the update step (referred to as IMM-update) of the IMM-KF using measurements from GPS, BD2 and CNS. Next, we present the proposed failure detection methodology.

### 3.2. Failure Detection Methodology

**Figure 4:**
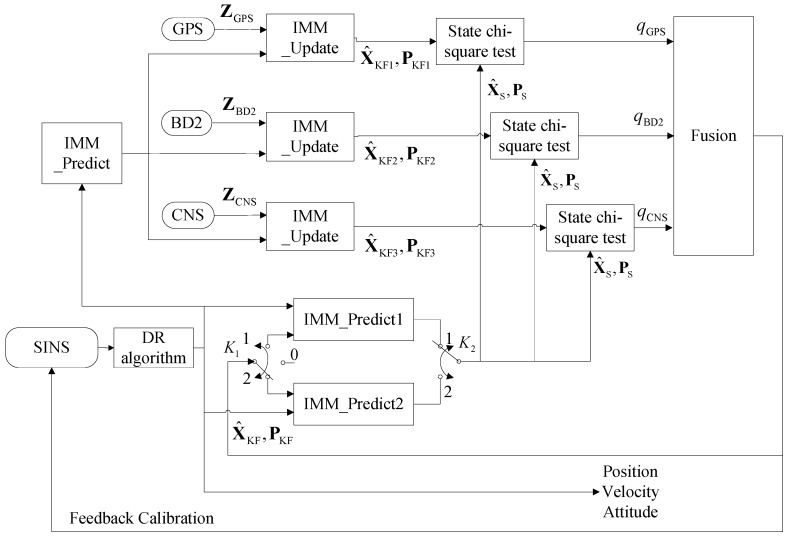
Block diagram of the failure detection method.

Figure 4 illustrates the block diagram of the proposed failure detection methodology which consists of three state $\chi^{2}$-test blocks. For presentation purposes, assume that the IMM-Predict 1 and IMM-Predict 2 provide the predicted state estimate ${\hat{x}}_{S}$ and its associated covariance matrix $P_{S}$, the IMM-update module provides the estimated state ${\hat{x}}_{\text{KF}}$ and its associated covariance matrix $P_{\text{KF}}$ after completion of the measurement update step. The failure detection function is defined as follows:

(12) $$\begin{array}{c} q=\frac{{\left({\hat{x}}_{\text{KF}}-{\hat{x}}_{S}\right)}^{T}\left({\hat{x}}_{\text{KF}}-{\hat{x}}_{S}\right)}{\left(P_{S}-P_{\text{KF}}\right)}∼\chi^{2}\left(n\right), \end{array}$$

where *n* is the dimension of the state vector. Failure of a measurement obtained from each sub-system is tested based on the following detection rule

(13) $$\begin{array}{c} \left\{\begin{array}{cc} \text{if}\;q\geq T_{D}, & \text{There is a measurement failure}.\\ \text{if}\;q<T_{D}, & \text{No measurement failure}. \end{array}\right. \end{array}$$

where the value of $T_{D}$ can be obtained from the table of $\chi^{2}$ [14]. Intuitively speaking, when the IMM filter is incorporated into the failure detection algorithm it would be hard to detect soft measurement failures. This is because the IMM filter updates the probability of each model using the measurement values which gets affected by measurement failures. We design the failure detection process to solve this problem. In this regard, first we define the following time-related variables: (i) the state propagators’ calibration cycle is denoted by Δt; (ii) the resolution cycle of the SINS is denoted by $T_{\text{SINS}}$; (iii) the measurement update cycle is denoted by $T_{2}$; and (iv) the feedback calibration cycle of the SINS is denoted by $T_{4}$, such that the following conditions are satisfied:

(14) $$\begin{array}{c} \Delta t>T_{2}>T_{\text{SINS}}>0,\;\text{and}\;\;T_{4}>T_{2}>T_{\text{SINS}}>0. \end{array}$$

For example, $T_{\text{SINS}}=0.01$, $T_{2}=1$ s, $T_{4}=60$ s and Δt=30 s. The failure detection process is defined in terms of these time-related variables and consists of the following steps:

**Step 1:**  System initialization: Let t=0, we switch $K_{1}$ to “0”, which means that we do not calibrate the state propagators. We switch $K_{2}$ to “1”, which means we use the ${\hat{x}}_{S}$ and $P_{S}$ provided from the “IMM-Predict 1” sub-system as the test reference.
**Step 2:**  If $t=n_{1}T_{\text{SINS}}$, for $1\leq n_{1}\leq n$, then we conduct the dead-reckoning algorithm of the SINS (denoted as the DR algorithm in Figure 4).
**Step 3:**  If $t=n_{2}T_{2}$, for $1\leq n_{2}\leq n$, then we update the sub-system with the process “IMM-update", and output the ${\hat{x}}_{\text{KF}}$ and $P_{\text{KF}}$ with each sub-system. We use Equation (12) to calculate the failure detection value *q* and send *q*, ${\hat{x}}_{\text{KF}}$ and $P_{\text{KF}}$ obtained from each sub-system to the fusion module to conduct failure isolation and state fusion.
**Step 4:**  If $t=n_{3}\Delta t$, for $1\leq n_{3}\leq n$, and if $n_{3}=2i+1$, for (1≤i≤n), we switch $K_{1}$ to “1”, and let ${\hat{x}}_{k-1|k-1}^{j}={\hat{x}}_{\text{KF}}^{j}$, $P_{k-1|k-1}^{j}=P_{\text{KF}}^{j}$ and $\mu^{j}=\mu_{\text{KF}}^{j}$, which means that we calibrate the state propagators of “IMM-Predict 1” with the fusion output. Meanwhile, we switch $K_{2}$ to “2”, which means we use the ${\hat{x}}_{S}$ and $P_{S}$ outputs from “IMM-Predict 2” as the test reference. If $n_{3}=2i$, we switch $K_{1}$ to “2”, which means that we calibrate the state propagators of “IMM-Predict 2” with the fusion output. Meanwhile, we switch $K_{2}$ to “1”, to use the ${\hat{x}}_{S}$ and $P_{S}$ outputs from “IMM-Predict 1” as the test reference.
**Step 5:**  If $t=n_{4}T_{4}$, for $1\leq n_{4}\leq n$, we conduct feedback calibration to the SINS.
**Step 6:**  Finally, when $t=t+T_{\mathrm{SINS}}$, if the system is powered on switch to “Step 2”, otherwise, terminate.

**Figure 5:**
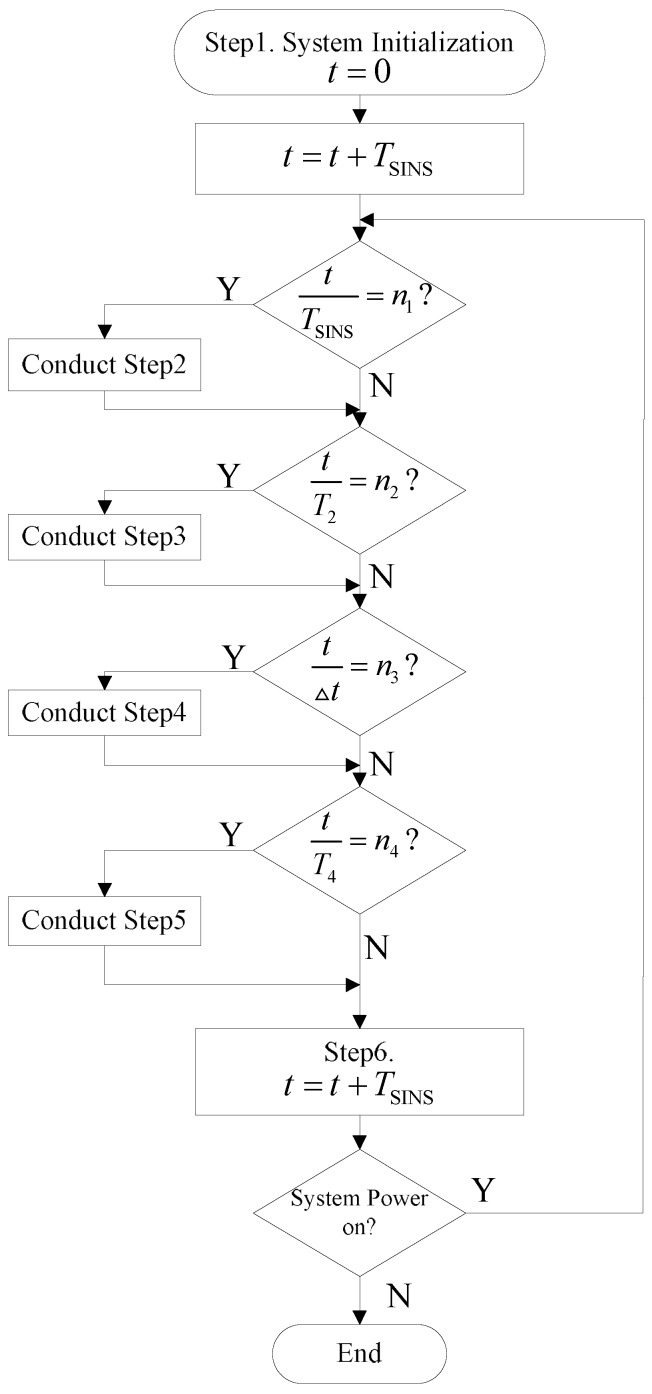
Algorithm flowchart.

We note that the above algorithm will run based on the inertial measurement units’ sampling time. In practice, this means that the algorithm will run as the system is powered on, therefore, as time increases, the algorithm will run step by step until the system is powered off. Figure 5 illustrates the flowchart of this algorithm.

Through implementation of the above proposed fault detection approach, we eliminate the risk of updating the probability of each model using contaminated measurements which in turn increases the failure detection sensitivity of the proposed methodology. Next, we present the fusion framework which uses the result from local IMM-KFs together with the fault detection module to form the overall state estimate and its corresponding error covariance matrix at each filtering iteration.

### 3.3. Information Fusion Framework

In order to combine local estimation results obtained from each sub-system, we expand the fusion method proposed in [19] to the SINS/GPS/BD2/CNS integrated navigation system incorporating our development in Section 3.1 and Section 3.2. The modified fusion method is used for isolating measurement failures and fusing state estimates. Besides, information from failure detection modules and measurement update information are used to choose a proper measurement update for the IMM algorithm for updating the probability of each model. The proposed fusion framework computes the overall state estimate and its associated covariance matrix, at each iteration, by forming a weighted average of the state estimates obtained from each local IMM-KF matched to a specific navigation sub-system. The adaptive weights used to fuse local state estimates to form the overall state estimate are defined based on the validation probabilities corresponding to each navigation sub-system as described below. In order to develop the proposed fusion methodology, we define the validation probability of each measurement according to the following fuzzy logic:

(15) $$\begin{array}{c} \beta =\left\{\begin{array}{cc} 1 & q\leq 6.25\\ -\frac{q}{5.1}+\frac{11.35}{5.1} & 6.25<q\leq 11.35.\\ 0 & q>11.35 \end{array}\right. \end{array}$$

**Figure 6:**
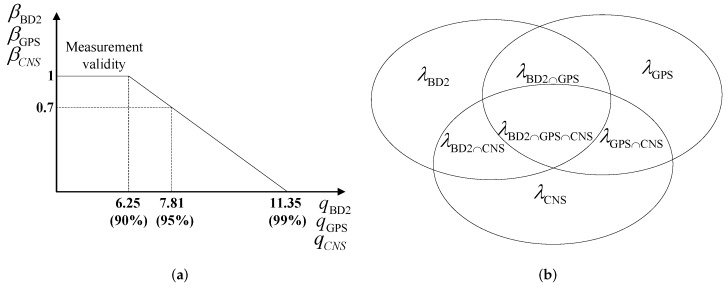
(**a**) Measurement probability of validity; (**b**) Probability space.

The quadratic form $q\in R^{+}$ defined in Equation (12) is theoretically a $\chi^{2}$ distribution with three degrees of freedom [14]. From standard $\chi^{2}$ tables, it is possible to define the validity domains of the each sensor’s measurement depending on the required confidence level, i.e., if the value of *q* is beyond a predefined threshold $T_{D}$, then it is assumed that the sensor has failed. In practice, however, it is hard to have a predefined threshold; therefore, we proposed the fuzzy logic solution. From the standard $\chi^{2}$ tables, considering a 90% confidence level and noting that the dimension is three, then $T_{D}=6.25$. On the other hand, considering a 99% confidence level, then $T_{D}=11.35$. This implies that when q≤6.25, the sensor is definitely reliable while when q>11.35, the sensor has failed. Finally, when 6.25<q≤11.35, the sensor is between the state of failure and being reliable. Equation (15) defines the probability of validity as described above. As Figure 6a illustrates, the validation probability of each measurement is a function of its corresponding failure detection value *q*. Based on the validation probability given in Equation (15), the validation probability of each sub-system’s measurement and the validation probability of the integrated sub-systems are defined as follows:

(16) $$\;\left\{\begin{array}{c} \lambda_{\text{BD}2}=\beta \left(q_{\text{BD}2}\right)\\ \lambda_{\text{GPS}}=\beta \left(q_{\text{GPS}}\right)\\ \lambda_{\text{CNS}}=\beta \left(q_{\text{CNS}}\right)\\ \lambda_{\text{BD}2\cap \text{GPS}}=\beta \left(q_{\text{BD}2}\right)\beta \left(q_{\text{GPS}}\right)\\ \lambda_{\text{BD}2\cap \text{CNS}}=\beta \left(q_{\text{BD}2}\right)\beta \left(q_{\text{CNS}}\right)\\ \lambda_{\text{GPS}\cap \text{CNS}}=\beta \left(q_{\text{GPS}}\right)\beta \left(q_{\text{CNS}}\right)\\ \lambda_{\text{BD}2\cap \text{GPS}\cap \text{CNS}}=\beta \left(q_{\text{BD}2}\right)\beta \left(q_{\text{GPS}}\right)\beta \left(q_{\text{CNS}}\right) \end{array}\right.$$

where $\lambda_{\text{BD}2}$ denotes the probability that BD2 sub-system is valid. Similarly, $\lambda_{\text{GPS}}$ and $\lambda_{\text{CNS}}$ denote the validation probability of GPS and CNS sub-systems, respectively. Term $\lambda_{\text{BD}2\cap \text{GPS}}$ provides the probability that both BD2 and GPS sub-systems are valid at the same time, while $\lambda_{\text{BD}2\cap \text{CNS}}$ corresponds to the scenario where BD2 and CNS sub-systems are valid at the same time, and $\lambda_{\text{GPS}\cap \text{CNS}}$ denotes the probability that GPS and CNS sub-systems are valid at the same time. Finally, $\lambda_{\text{BD}2\cap \text{GPS}\cap \text{CNS}}$ defines the probability that all three subsystems (BD2, GPS and CNS) are valid at the same time.

Figure 6b shows, graphically, the probability space specified above. Based on the aforementioned probability space, the weight of each sub-system is calculated as follows

(17) $$\left\{\begin{array}{c} \alpha_{\text{BD}2}=\lambda_{\text{BD}2}-\lambda_{\text{BD}2\cap \text{GPS}}-\lambda_{\text{BD}2\cap \text{CNS}}+\lambda_{\text{BD}2\cap \text{GPS}\cap \text{CNS}}\\ \alpha_{\text{GPS}}=\lambda_{\text{GPS}}-\lambda_{\text{BD}2\cap \text{GPS}}-\lambda_{\text{GPS}\cap \text{CNS}}+\lambda_{\text{BD}2\cap \text{GPS}\cap \text{CNS}}\\ \alpha_{\text{CNS}}=\lambda_{\text{CNS}}-\lambda_{\text{BD}2\cap \text{CNS}}-\lambda_{\text{GPS}\cap \text{CNS}}+\lambda_{\text{BD}2\cap \text{GPS}\cap \text{CNS}}\\ \alpha_{\text{GPS}}=\lambda_{\text{GPS}}-\lambda_{\text{BD}2\cap \text{GPS}}-\lambda_{\text{GPS}\cap \text{CNS}}+\lambda_{\text{BD}2\cap \text{GPS}\cap \text{CNS}}\\ \alpha_{\text{BD}2+\text{GPS}}=\lambda_{\text{BD}2\cap \text{GPS}}-\lambda_{\text{BD}2\cap \text{GPS}\cap \text{CNS}}\\ \alpha_{\text{BD}2+\text{CNS}}=\lambda_{\text{BD}2\cap \text{CNS}}-\lambda_{\text{BD}2\cap \text{GPS}\cap \text{CNS}}\\ \alpha_{\text{GPS}+\text{CNS}}=\lambda_{\text{GPS}\cap \text{CNS}}-\lambda_{\text{BD}2\cap \text{GPS}\cap \text{CNS}}\\ \alpha_{\text{BD}2+\text{GPS}+\text{CNS}}=\lambda_{\text{BD}2\cap \text{GPS}\cap \text{CNS}}\\ \alpha_{\text{SINS}}=1-\lambda_{\text{BD}2}-\;\lambda_{\text{GPS}}-\;\lambda_{\text{CNS}}-\;\lambda_{\text{BD}2\cap \text{GPS}}-\;\lambda_{\text{BD}2\cap \text{CNS}}-\;\lambda_{\text{GPS}\cap \text{CNS}}-\;\lambda_{\text{BD}2\cap \text{GPS}\cap \text{CNS}} \end{array}\right.$$

where $\alpha_{\text{BD}2}$ refers to the scenario where only the BD2 sub-system is valid. Similarly, $\alpha_{\text{GPS}}$ means that only the GPS sub-system is valid, and $\alpha_{\text{CNS}}$ means that only the CNS sub-system is valid. On the other hand, $\alpha_{\text{BD}2+\text{GPS}}$ means that the BD2 and the GPS sub-systems are both valid at the same time; $\alpha_{\text{BD}2+\text{CNS}}$ means that the BD2 and the CNS sub-systems are both valid at the same time; and term $\alpha_{\text{GPS}\cap \text{CNS}}$ means that the GPS and the CNS sub-systems are valid at the same time. Term $\alpha_{\text{BD}2+\text{GPS}+\text{CNS}}$ means that all three sub-systems (BD2, GPS and CNS) are valid at the same time, while finally, term $\alpha_{\text{SINS}}$ means that all three local sub-systems (the BD2, GPS and CNS) have failed at the same time.

The proposed fusion framework computes the overall state estimate and its associated covariance matrix at each iteration by forming a weighted average of the estimates computed from each navigation sub-system, where the weights are the validation probabilities defined above. The state ${\hat{x}}_{k|k}^{\left(\text{GPS}\right)}$ and covariance matrix $P_{k|k}^{\left(\text{GPS}\right)}$ corresponding to the stand-alone scenario based on GPS data are computed from the “IMM-update” of the local IMM-KF matched to the GPS signal as explained previously in Section 3.1. Similarly, the state and covariance matrix (${\hat{x}}_{k|k}^{\left(\text{BD}2\right)}$ and $P_{k|k}^{\left(\text{BD}2\right)}$) corresponding to the stand-alone scenario based on the BD2 data and the ones (${\hat{x}}_{k|k}^{\left(\text{CNS}\right)}$ and $P_{k|k}^{\left(\text{CNS}\right)}$) corresponding to the stand-alone scenario based on the CNS data are computed from the “IMM-update” of the local IMM-KF matched to the BD2 and the CNS signals, respectively. The fusion algorithm besides the above estimates also needs the estimates for the remaining four scenarios where instead of having a stand-alone sub-system two or all three of the sub-systems are active (no failure). In the case that the BD2 and the GPS sub-systems are valid at the same time, the state estimate and its corresponding covariance matrix are computed as follows:

(18) $$\begin{array}{cc} {\hat{x}}_{k|k}^{\left(\text{BD}2+\text{GPS}\right)} & ={\hat{x}}_{k|k-1}+K_{k}^{\left(\text{BD}2|\text{BD}2+\text{GPS}\right)}(z_{k}^{\left(\text{BD}2\right)}-H^{\left(\text{BD}2\right)}{\hat{x}}_{k|k-1})\\ & +K_{k}^{\left(\text{GPS}|\text{BD}2+\text{GPS}\right)}(z_{k}^{\left(\text{GPS}\right)}-H^{\left(\text{GPS}\right)}{\hat{x}}_{k|k-1}) \end{array}$$

(19) $${\left[P_{k|k}^{\left(\text{BD}2+\text{GPS}\right)}\right]}^{-1}=P_{k|k-1}^{-1}+{\left[H^{\left(\text{BD}2\right)}\right]}^{T}{\left[R^{\left(\text{BD}2\right)}\right]}^{-1}H^{\left(\text{BD}2\right)}+{\left[H^{\left(\text{GPS}\right)}\right]}^{T}{\left[R^{\left(\text{GPS}\right)}\right]}^{-1}H^{\left(\text{GPS}\right)}$$

where the Kalman gains are given by

(20) $$\begin{array}{ccc} K_{k}^{\left(\text{BD}2|\text{BD}2+\text{GPS}\right)} & = & P_{k|k}^{\left(\text{BD}2+\text{GPS}\right)}{\left[H^{\left(\text{BD}2\right)}\right]}^{T}{\left[R^{\left(\text{BD}2\right)}\right]}^{-1} \end{array}$$

(21) $$\begin{array}{ccc} K_{k}^{\left(\text{GPS}|\text{BD}2+\text{GPS}\right)} & = & P_{k|k}^{\left(\text{BD}2+\text{GPS}\right)}{\left[H^{\left(\text{GPS}\right)}\right]}^{T}{\left[R^{\left(\text{GPS}\right)}\right]}^{-1}. \end{array}$$

On the other hand, when the BD2 and the CNS sub-systems are valid at the same time, the state estimate and its corresponding covariance matrices are given by

(22) $$\begin{array}{cc} {\hat{x}}_{k|k}^{\left(\text{BD}2+\text{CNS}\right)} & ={\hat{x}}_{k|k-1}+K_{k}^{\left(\text{BD}2|\text{BD}2+\text{CNS}\right)}(z_{k}^{\left(\text{BD}2\right)}-H^{\left(\text{BD}2\right)}{\hat{x}}_{k|k-1})\\ & +K_{k}^{\left(\text{CNS}|\text{BD}2+\text{CNS}\right)}(z_{k}^{\left(\text{CNS}\right)}-H^{\left(\text{CNS}\right)}{\hat{x}}_{k|k-1}) \end{array}$$

(23) $${\left[P_{k|k}^{\left(\text{BD}2+\text{CNS}\right)}\right]}^{-1}=P_{k|k-1}^{-1}+{\left[H^{\left(\text{BD}2\right)}\right]}^{T}{\left[R^{\left(\text{BD}2\right)}\right]}^{-1}H^{\left(\text{BD}2\right)}+{\left[H^{\left(\text{CNS}\right)}\right]}^{T}{\left[R^{\left(\text{CNS}\right)}\right]}^{-1}H^{\left(\text{CNS}\right)},$$

where the Kalman gains are computed as

(24) $$\begin{array}{ccc} K_{k}^{\left(\text{BD}2|\text{BD}2+\text{CNS}\right)} & = & P_{k|k}^{\left(\text{BD}2+\text{CNS}\right)}{\left[H^{\left(\text{BD}2\right)}\right]}^{T}{\left[R^{\left(\text{BD}2\right)}\right]}^{-1} \end{array}$$

(25) $$\begin{array}{ccc} K_{k}^{\left(\text{CNS}|\text{BD}2+\text{CNS}\right)} & = & P_{k|k}^{\left(\text{BD}2+\text{CNS}\right)}{\left[H^{\left(\text{CNS}\right)}\right]}^{T}{\left[R^{\left(\text{CNS}\right)}\right]}^{-1}. \end{array}$$

In the case where the GPS and the CNS sub-systems are valid at the same time, the state estimate and covariance update are as follows

(26) $$\begin{array}{cc} {\hat{x}}_{k|k}^{\left(\text{GPS}+\text{CNS}\right)} & ={\hat{x}}_{k|k-1}+K_{k}^{\left(\text{GPS}|\text{GPS}+\text{CNS}\right)}(z_{k}^{\left(\text{GPS}\right)}-H^{\left(\text{GPS}\right)}{\hat{x}}_{k|k-1})\\ & +K_{k}^{\left(\text{CNS}|\text{GPS}+\text{CNS}\right)}(z_{k}^{\left(\text{CNS}\right)}-H^{\left(\text{CNS}\right)}{\hat{x}}_{k|k-1}) \end{array}$$

(27) $${\left[P_{k|k}^{\left(\text{GPS}+\text{CNS}\right)}\right]}^{-1}=P_{k|k-1}^{-1}+{\left[H^{\left(\text{GPS}\right)}\right]}^{T}{\left[R^{\left(\text{GPS}\right)}\right]}^{-1}H^{\left(\text{GPS}\right)}+{\left[H^{\left(\text{CNS}\right)}\right]}^{T}{\left[R^{\left(\text{CNS}\right)}\right]}^{-1}H^{\left(\text{CNS}\right)},$$

where the Kalman gains are given by

(28) $$\begin{array}{ccc} K_{k}^{\left(\text{GPS}|\text{GPS}+\text{CNS}\right)} & = & P_{k|k}^{\left(\text{GPS}+\text{CNS}\right)}{\left[H^{\left(\text{GPS}\right)}\right]}^{T}{\left[R^{\left(\text{GPS}\right)}\right]}^{-1} \end{array}$$

(29) $$\begin{array}{ccc} K_{k}^{\left(\text{CNS}|\text{GPS}+\text{CNS}\right)} & = & P_{k|k}^{\left(\text{GPS}+\text{CNS}\right)}{\left[H^{\left(\text{CNS}\right)}\right]}^{T}{\left[R^{\left(\text{CNS}\right)}\right]}^{-1}. \end{array}$$

Finally, when the BD2, the GPS, and the CNS sub-systems are all valid, the state estimate and its associated covariance matrix are given by

(30) $$\begin{array}{ccc} {\hat{x}}_{k|k}^{\left(\text{BD}2+\text{GPS}+\text{CNS}\right)} & = & {\hat{x}}_{k|k-1}+K_{k}^{\left(\text{BD}2|\text{BD}2+\text{GPS}+\text{CNS}\right)}(z_{k}^{\left(\text{BD}2\right)}-H^{\left(\text{BD}2\right)}{\hat{x}}_{k|k-1})\\ & + & K_{k}^{\left(\text{GPS}|\text{BD}2+\text{GPS}+\text{CNS}\right)}(z_{k}^{\left(\text{GPS}\right)}-H^{\left(\text{GPS}\right)}{\hat{x}}_{k|k-1})\\ & + & K_{k}^{\left(\text{CNS}|\text{BD}2+\text{GPS}+\text{CNS}\right)}(z_{k}^{\left(\text{CNS}\right)}-H^{\left(\text{CNS}\right)}{\hat{x}}_{k|k-1}) \end{array}$$

(31) $$\begin{array}{cc} {\left[P_{k|k}^{\left(\text{BD}2+\text{GPS}+\text{CNS}\right)}\right]}^{-1} & =P_{k|k-1}^{-1}+{\left[H^{\left(\text{BD}2\right)}\right]}^{T}{\left[R^{\left(\text{BD}2\right)}\right]}^{-1}H^{\left(\text{BD}2\right)}\\ & +{\left[H^{\left(\text{GPS}\right)}\right]}^{T}{\left[R^{\left(\text{GPS}\right)}\right]}^{-1}H^{\left(\text{GPS}\right)}+{\left[H^{\left(\text{CNS}\right)}\right]}^{T}{\left[R^{\left(\text{CNS}\right)}\right]}^{-1}H^{\left(\text{CNS}\right)}, \end{array}$$

where the Kalman gains are computed as follows

(32) $$\begin{array}{ccc} K_{k}^{\left(\text{BD}2|\text{BD}2+\text{GPS}+\text{CNS}\right)} & = & P_{k|k}^{\left(\text{BD}2+\text{GPS}+\text{CNS}\right)}{\left[H^{\left(\text{BD}2\right)}\right]}^{T}{\left[R^{\left(\text{BD}2\right)}\right]}^{-1} \end{array}$$

(33) $$\begin{array}{ccc} K_{k}^{\left(\text{GPS}|\text{BD}2+\text{GPS}+\text{CNS}\right)} & = & P_{k|k}^{\left(\text{BD}2+\text{GPS}+\text{CNS}\right)}{\left[H^{\left(\text{GPS}\right)}\right]}^{T}{\left[R^{\left(\text{GPS}\right)}\right]}^{-1} \end{array}$$

(34) $$\begin{array}{ccc} K_{k}^{\left(\text{CNS}|\text{BD}2+\text{GPS}+\text{CNS}\right)} & = & P_{k|k}^{\left(\text{BD}2+\text{GPS}+\text{CNS}\right)}{\left[H^{\left(\text{CNS}\right)}\right]}^{T}{\left[R^{\left(\text{CNS}\right)}\right]}^{-1}. \end{array}$$

Based on the above set of local state estimates, the final state estimate and its corresponding error covariance matrix are given by

(35) $$\begin{array}{cc} {\hat{x}}_{k|k} & =\alpha_{\text{SINS}}{\hat{x}}_{k|k-1}\\ & +\alpha_{\text{BD}2}{\hat{x}}_{k|k}^{\left(\text{BD}2\right)}+\alpha_{\text{GPS}}{\hat{x}}_{k|k}^{\left(\text{GPS}\right)}+\alpha_{\text{CNS}}{\hat{x}}_{k|k}^{\left(\text{CNS}\right)}\\ & +\alpha_{\text{BD}2+\text{GPS}}{\hat{x}}_{k|k}^{\left(\text{BD}2+\text{GPS}\right)}+\alpha_{\text{BD}2+\text{CNS}}{\hat{x}}_{k|k}^{\left(\text{BD}2+\text{CNS}\right)}+\alpha_{\text{GPS}+\text{CNS}}{\hat{x}}_{k|k}^{\left(\text{GPS}+\text{CNS}\right)}\\ & +\alpha_{\text{BD}2+\text{GPS}+\text{CNS}}{\hat{x}}_{k|k}^{\left(\text{BD}2+\text{GPS}+\text{CNS}\right)} \end{array}$$

(36) $$\begin{array}{cc} P_{k|k} & =\alpha_{\text{SINS}}P_{k|k-1}\\ & +\alpha_{\text{BD}2}\left[P_{k|k}^{\left(\text{BD}2\right)}+\left({\hat{x}}_{k|k}-{\hat{x}}_{k|k}^{\left(\text{BD}2\right)}\right){\left({\hat{x}}_{k|k}-{\hat{x}}_{k|k}^{\left(\text{BD}2\right)}\right)}^{T}\right]\\ & +\alpha_{\text{GPS}}\left[P_{k|k}^{\left(\text{GPS}\right)}+\left({\hat{x}}_{k|k}-{\hat{x}}_{k|k}^{\left(\text{GPS}\right)}\right){\left({\hat{x}}_{k|k}-{\hat{x}}_{k|k}^{\left(\text{GPS}\right)}\right)}^{T}\right]\\ & +\alpha_{\text{CNS}}\left[P_{k|k}^{\left(\text{CNS}\right)}+\left({\hat{x}}_{k|k}-{\hat{x}}_{k|k}^{\left(\text{CNS}\right)}\right){\left({\hat{x}}_{k|k}-{\hat{x}}_{k|k}^{\left(\text{CNS}\right)}\right)}^{T}\right]\\ & +\alpha_{\text{BD}2+\text{GPS}}\left[P_{k|k}^{\left(\text{BD}2+\text{GPS}\right)}+\left({\hat{x}}_{k|k}-{\hat{x}}_{k|k}^{\left(\text{BD}2+\text{GPS}\right)}\right){\left({\hat{x}}_{k|k}-{\hat{x}}_{k|k}^{\left(\text{BD}2+\text{GPS}\right)}\right)}^{T}\right]\\ & +\alpha_{\text{BD}2+\text{CNS}}\left[P_{k|k}^{\left(\text{BD}2+\text{CNS}\right)}+\left({\hat{x}}_{k|k}-{\hat{x}}_{k|k}^{\left(\text{BD}2+\text{CNS}\right)}\right){\left({\hat{x}}_{k|k}-{\hat{x}}_{k|k}^{\left(\text{BD}2+\text{CNS}\right)}\right)}^{T}\right]\\ & +\alpha_{\text{GPS}+\text{CNS}}\left[P_{k|k}^{\left(\text{GPS}+\text{CNS}\right)}+\left({\hat{x}}_{k|k}-{\hat{x}}_{k|k}^{\left(\text{GPS}+\text{CNS}\right)}\right){\left({\hat{x}}_{k|k}-{\hat{x}}_{k|k}^{\left(\text{GPS}+\text{CNS}\right)}\right)}^{T}\right]\\ & +\alpha_{\text{BD}2+\text{GPS}+\text{CNS}}\left[P_{k|k}^{\left(\text{BD}2+\text{GPS}+\text{CNS}\right)}+\left({\hat{x}}_{k|k}-{\hat{x}}_{k|k}^{\left(\text{BD}2+\text{GPS}+\text{CNS}\right)}\right){\left({\hat{x}}_{k|k}-{\hat{x}}_{k|k}^{\left(\text{BD}2+\text{GPS}+\text{CNS}\right)}\right)}^{T}\right] \end{array}$$

To summarize, the iteration *k* of the fusion methodology is performed based on the following seven steps:

**Step 1:**  Conduct the “IMM-predict” process.
**Step 2:**  Conduct the “IMM-update” process. Here we use the following strategy to update the likelihood of the measurement. We compare the *q* of each sub-system, which sub-system has the minimum *q*, then we use the measurement of that sub-system to update the likelihood and thereby obtain the updated probability of the system models.
**Step 3:**  Calculate the failure detection value *q* using Equation (12) for each sub-system.
**Step 4:**  Calculate the probability of each measurement sub-system using Equations (15) and (16).
**Step 5:**  Calculate the weights of each sub-system using Equation (17).
**Step 6:**  Update the state and covariance of each sub-system using Equations (18)–(34).
**Step 7:**  Update the overall state estimate and its corresponding covariance matrix using Equations (35)–(36). This completes iteration *k* of the estimation algorithm.

Note that, the final results computed in Step 7 for iteration *k* are used as the initial values for the next iteration (k+1), and the estimation process continues sequentially as long as the system is powered on. This completes the development of the proposed multi-sensor fault tolerant information fusion framework, next we present our simulation results carried out to evaluate the performance of the proposed framework.

## 4. Results

**Figure 7:**
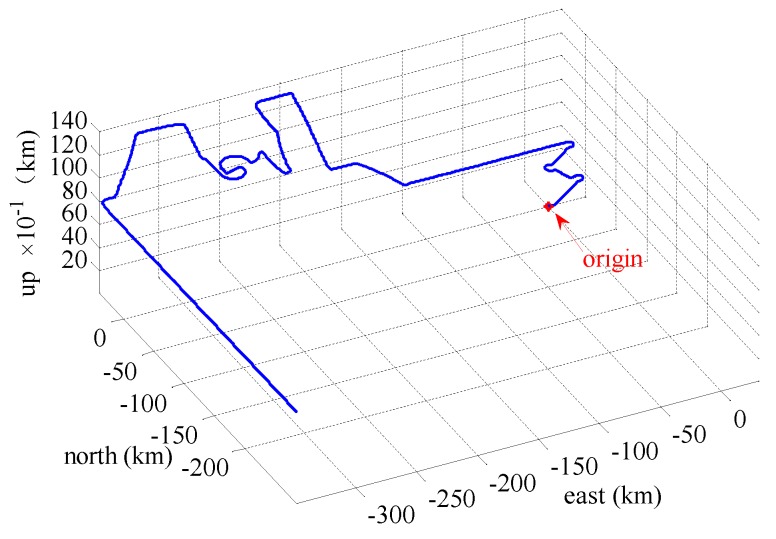
Aircraft trajectory.

**Figure 8:**
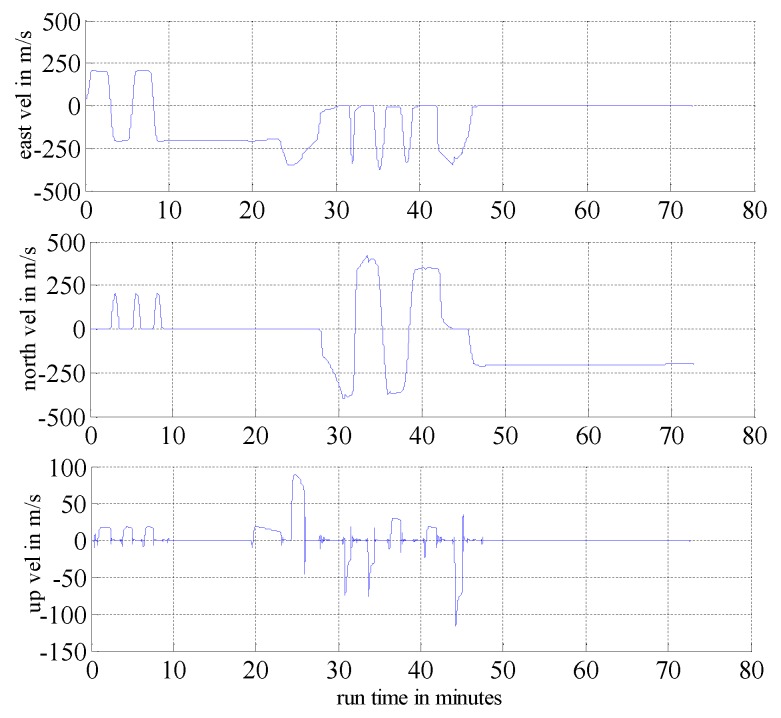
Aircraft velocity.

**Figure 9:**
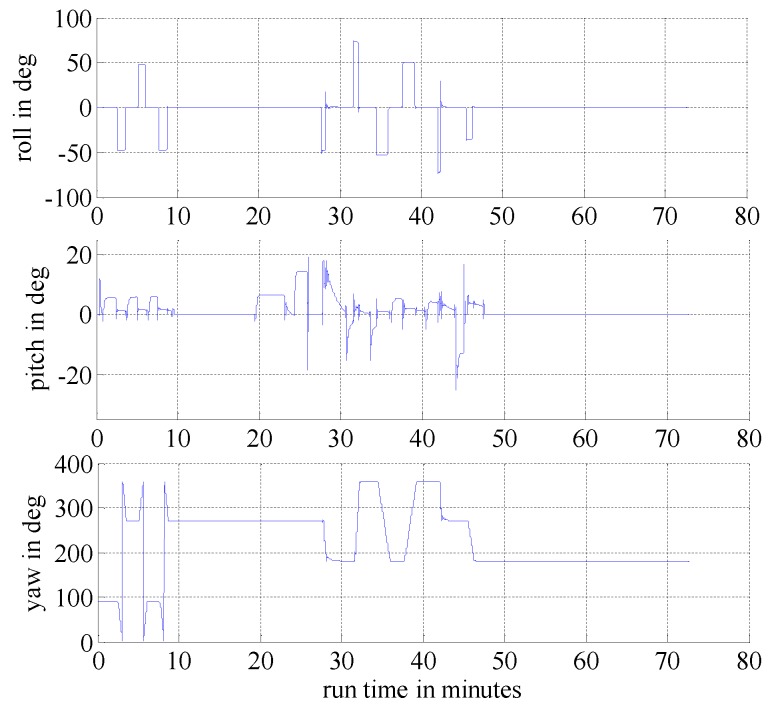
Aircraft attitude.

In this section, we present simulation examples carried out to evaluate the performance of the proposed multi-sensor and fault-tolerant fusion framework in comparison to its conventional counterparts. In these experiments, the aircraft trajectory and inertial measurements are generated using the “Inertial Navigation System toolbox” developed by GPSoft LLC [32]. Besides, the “Satellite Navigation toolbox” developed by GPSoft LLC [33] is employed to generate the BD2 and GPS positions. The attitude obtained by the CNS was generated by the commercial software “Inertial Navigation System toolbox” [32]. The aircraft trajectory is illustrated in Figure 7, while Figure 8 and Figure 9 show the aircraft velocity and attitude, respectively. From Figure 7, Figure 8 and Figure 9, we can see that from 0 ∼ 10 min, the aircraft is under a high manoeuvre. The aircraft is under a low manoeuvre between 10 ∼ 20 min, while it is considered to be under high manoeuvre between 20 ∼ 50 min. After the 50 min point, the aircraft remains under the low manoeuvre. Before presenting the results, we briefly outline the parameters used in the reported experiments in Table 1, Table 2 and Table 3. We note that, these parameters are selected to simulate real-world scenarios. Based on these parameters, in the following two sub-sections, we present two different simulation scenarios. The first scenario examines the situation where the accuracy of the SINS sub-system is relatively low. In the second scenario, we evaluate the performance of the proposed detection algorithm in scenarios with multiple failures. The simulation experiments have been performed on a PC computer with the following specification: CPU: Intel core i5 3320M, 2.6 GHz; RAM: 4GB 1333 Hz. Based on this available computational power, each algorithm cycle takes 0.0053 s at the maximum. In practical settings of the problem at hand, the sample cycle is typically 100 Hz which means that the complexity of the proposed framework would not be an issue. In other words, factoring in the reliability and fault-tolerant performance of the proposed system, the computational cost is reasonably acceptable.

### 4.1. Scenario 1

**Figure 10:**
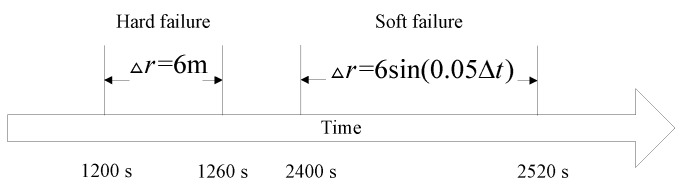
Failure timing sequence.

**Figure 11:**
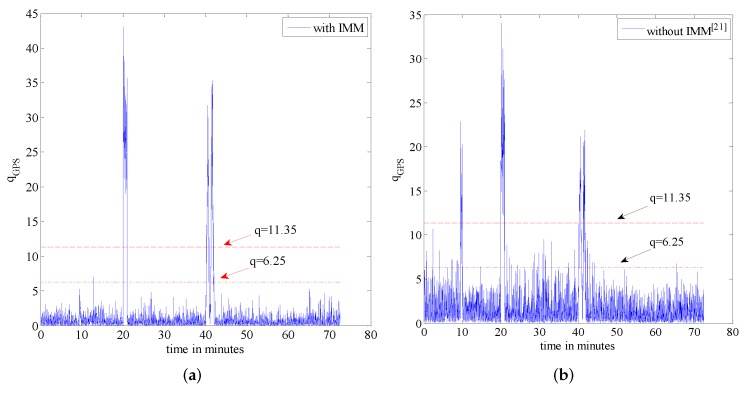
Failure detection curve. (**a**) Algorithm with IMM; (**b**) Algorithm without IMM [20].

As stated previously, in this scenario, the accuracy of the SINS is simulated in low settings; therefore, its position accuracy will be affected adversely by the aircraft manoeuvres. In this scenario, we compare the proposed fault tolerant algorithm with its conventional counterpart without the incorporation of an IMM filtering algorithm, which is proposed in [20]. For completeness, we evaluate potential performance improvements both in terms of the reduction achieved in the false alarm rate and improvements observed in failure detection sensitivity. We add the step change with an amplitude of 6 m to simulate the hard failure situation in the GPS signal and add the sine change to simulate the soft failure of the GPS signal. Figure 10 shows the failure timing sequence used in the simulations. For the two models used in the IMM step, two separate process noise covariance matrices are considered. Model 1 represents the system with the smaller process noise denoted by $Q_{\text{small}}$, while the system with larger process noise, denoted by $Q_{\text{large}}$, is represented by Model 2. In this paper, the ratio of the process noise is considered to be 100, i.e., $Q_{\text{large}}=100\times Q_{\text{small}}$. The model transition probability matrix governing the switching points is considered as $p_{ij}=\left[\begin{array}{cc} 0.98 & 0.02\\ 0.02 & 0.98 \end{array}\right]$. Finally, the initial model probability is considered to be $\mu ={\left[0.9\;0.1\right]}^{T}$. Figure 11 shows the failure detection curve of the proposed algorithm and the one obtained from the conventional solution without the implementation of the IMM step. Based on Figure 11, it is observed that the proposed fault-tolerant fusion algorithm which utilizes the IMM step in its design outperforms its counterpart. It is also observed that the conventional solution without using an IMM step fails to produces reasonable results. The reason behind this observation is that the accuracy of the SINS simulated here is low; therefore, its output degrades by the aircraft manoeuvres. As such, the conventional solution is incapable of handling this situation because of the mismatch in the system’s model, i.e., the assumed system becomes different form the actual model. Note that the mismatch occurs as aircraft manoeuvres can change the model of the system. On the other hand, the proposed fusion framework, which uses two models to better approach the real scenario, performs well, as it can adjust to system’s uncertainty adaptively over time. Incorporation of the IMM-like step in the proposed framework improves the accuracy of state propagators, thereby increasing the sensitivity of the failure detection solution in the process. From Figure 11, it is also observed that in the absence of the IMM step and with low accuracy SINS, soft failures are hard to detect.

Table 4 shows the false alarm obtained from each of the two implemented algorithms. Similarly, Table 5 illustrates the failure detection time corresponding to the two implemented estimation algorithms. From Table 4, it is observed that incorporation of the IMM filtering step significantly reduces the false alarm rate. From Table 5, it is observed that although the algorithm without using the IMM step can detect hard failures reasonably well, it fails to detect soft failures, as was expected. We note that the soft failure detection time only shows the first half of the cycle of the sine failure. The time for the algorithm without the incorporation of an IMM step is not shown here, as the failure detection without IMM fails to work properly in this situation. In other words, as can be observed from Figure 11, the algorithm without using the IMM fails to detect soft failures with a high false alarm rate, which makes it hard to decide when it failed or not based on the data.

### 4.2. Scenario 2

**Figure 12:**
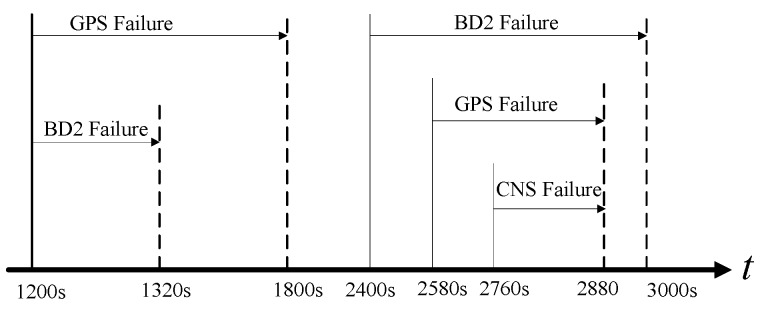
Failure timing sequence.

**Figure 13:**
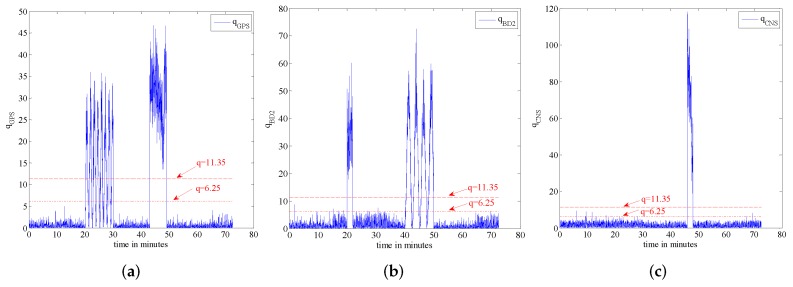
Failure detection (**a**) GPS failure; (**b**) GPS failure; (**c**) GPS failure.

**Figure 14:**
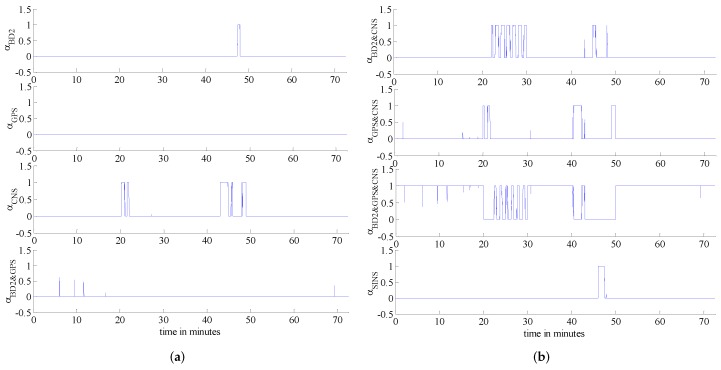
Sub-system weights (**a**) weights1; (**b**) weights2.

In order to further verify the validity of the proposed framework, we add extra failures as shown in Figure 12. The reason behind this experiment is to better verify the validity of our claim that the proposed fault-tolerant fusion framework is capable of detecting multiple failures happening at the same time. Table 6 outlines failure information used in this scenario in detail while the failure detection results are plotted in Figure 13. Figure 14 shows the weights of each sub-system calculated based on Equation (17). Based on Figure 13 and Figure 14, it is observed that the proposed fault-tolerant fusion framework, which utilizes the IMM step, can effectively detect failures and realize failure isolation. For example, between 0 min and 20 min, the three measurement sub-systems work properly, therefore, the weights obtained from the BD2, GPS and CNS are all one, which implies that the integrated system is SINS/GPS/BD2/CNS. On the other hand, within 20 min–22 min, the BD2 sub-system fails, and the GPS sub-system is in soft failure; therefore, the integrated system is switched between SINS/CNS and SINS/GPS/CNS. This can be observed from Figure 14. Between 46 min and 48 min, all three measurement subs-systems have failed, so only the weight of SINS is equal to one, which means that only the SINS works properly. Finally, between 0 min and 20 min, it is also observed from Figure 14 that there is some changes between different integrated systems; this is because the algorithm also has a false alarm; however, the false alarm is small and does not affect the overall performance of the system.

**Figure 15:**
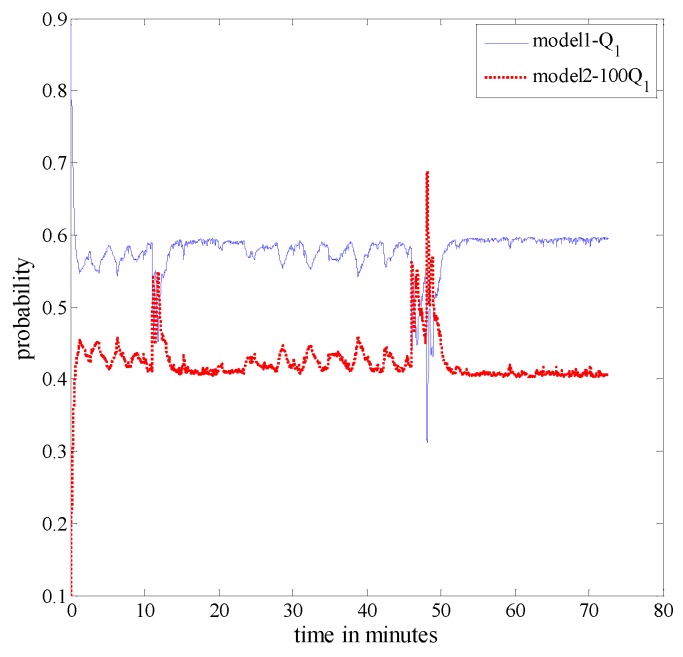
Probability of each model.

**Figure 16:**
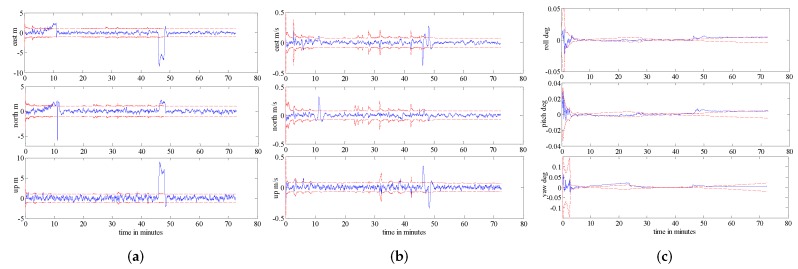
Error plots (**a**) Position error; (**b**) Velocity error; (**c**) Velocity error.

Figure 15 shows the probability of each model in the IMM algorithm. From Figure 15, we can see clear changes in the probability of each model between about 46 min ∼ 48 min. This is because all three measurements failed during this period; therefore, the system can only adjust adaptively through model probabilities. Figure 16 shows the estimation results corresponding to the position, velocity and attitude obtained from the proposed algorithm. From Figure 16, it is observed that between 20 min and 46 min, when the aircraft is under a high manoeuvre, the covariance of each state variable can change adaptively. This is because the proposed fusion framework, which uses two models to better approach the real scenario, performs superbly as it can adjust to the system’s uncertainties adaptively over time. From Figure 15, we can also observe that the probability of each model is not equal to one; this is because the real known model of the navigation system is hard to identify, and one can only obtain a set of candidate models to represent different uncertainties under realistic conditions. In other words, a trade-off is made when the system is under different manoeuvres. Between 46 min and 48 min, all three measurement sub-systems have failed, i.e., there is no more measurement to update the system; therefore, the error of the SINS will increase over time. This can also be observed from Figure 16. However, when measurement sub-systems return to work, the accuracy of the integrated navigation system returns promptly to the normal operation. In summary, the comparisons reported in this section are mainly with respect to [20,21] where a similar multi-sensor setup based on a SINS/GPS/BD2/CNS integrated navigation system is considered. The two scenarios considered in the paper are reported to achieve the following goals:

(1) In Scenario 1, the goal is to evaluate the potential achievable performance improvements of the proposed methodology both in terms of the reduction in the false alarm rate and improvements in failure detection sensitivity. In this regard, we present comparison results between the proposed algorithm and [20,21] to verify the validity of the proposed fault detection algorithm. It is observed that when we lower the accuracy of the inertial navigation unit, the algorithm proposed in [21] fails to work properly. This is mainly because of the absence of a precisely known model of the system in advance. Furthermore, it is also observed that the algorithm proposed in [20] performs poorly and provides the worst result.
(2) In Scenario 2, the goal is to verify the validity of the proposed fault-tolerant fusion framework in the presence of multi-sensor failures. In this case, both algorithms from [20,21] fail to provide acceptable results, and even the computed estimates based on these algorithms diverge from the ground truth. We note that the shortcoming of these algorithms in handling this practical scenario is due to the presence of soft failures and high aircraft manoeuvres.

## 5. Conclusions

The paper develops an innovative multi-sensor fault-tolerant fusion framework applied in the INS/GPS/BD2/CNS integrated navigation systems. We define two-level process noise covariance to represent uncertainties in the system’s model under low and high manoeuvres, with ideas from the IMM filtering domain. The state estimates obtained from the prediction step of the IMM filter, without the incorporation of the measurement update, are used as fault detection references. Consequently, the system’s uncertainty is adjusted adaptively by the model probabilities with the aircraft manoeuvre. In order to avoid the risk of using a contaminated state propagator as a fault detection reference, two IMM predictors, running in parallel, are used as the state propagators. These two state propagators are alternatively being reactivated with the information they receive from the fusion filter to increase their accuracy and, thereby, increase the fault sensitivity of the overall detection system. Finally, a fusion strategy is investigated to realize fault isolation where in order to use appropriate measurements for updating model probabilities, we take advantage of the fault detection information of each measurement. In other words, the measurement with the minimum fault detection value is utilized to update the model probabilities of the local IMM filters. This results in further improvements in the accuracy of the system as the model probabilities are updated based on the optimal measurement. Simulation results indicate that the proposed fault tolerant fusion framework provides improved performance over its traditional counterparts.

## Figures and Tables

**Table 1:** Biases and power spectra of the SINS sensors.

Sensor Parameter	Unit	Value
Accelerometer bias	μg	60
Accelerometer white noise	μg/$\sqrt{\text{Hz}}$	10
Gyro bias	deg/hour	1
Gyro white noise	deg/$\sqrt{\text{hour}}$	0.01

**Table 2:** Error of measurement.

Sensor Parameter	Unit	Value
GPS position error (longitude)	meter	2.92
GPS position error (latitude)	meter	3.06
GPS position error (vertical)	meter	2.98
BD2 position error (longitude)	meter	2.50
BD2 position error (latitude)	meter	2.75
BD2 position error (vertical)	meter	2.78
CNS measure error	arcseconds	3

**Table 3:** Initial error.

Sensor Parameter	Unit	Value
SINS body-x tilt error	rad	0.0001
SINS body-y tilt error	rad	0.0001
SINS body-z tilt error	rad	0.001
Initial east velocity error	m/s	0.02
Initial north velocity error	m/s	0.02
Initial up velocity error	m/s	0.02

**Table 4:** False alarm of the two algorithms.

Algorithm	False Alarm
Algorithm without IMM [20]	40%
Algorithm with IMM	0.72%

**Table 5:** Initial error.

Algorithm	Failure Type	Detection Time
With IMM	Hard failure	Start: 1202 s
		End: 1260 s
Without IMM [20]	Hard failure	Start: 1202 s
		End: 1259 s
With IMM	Soft failure	Start: 2410 s
		End: 2451 s
Without IMM [20]	Soft failure	-
		-

**Table 6:** Initial error.

Algorithm	Failure Type	Failure Time
GPS	7sin(0.04Δt)m	1200 s ∼ 1800 s
	7.5	2580 s ∼ 2940 s
BD2	6.5	1200 s ∼ 1320 s
	8sin(0.02Δt)m	2400 s ∼ 3000 s
CNS	3”	2760 s ∼ 2880 s
